# Supplementary material for: Neuron–Microglia Contact-Dependent Mechanisms Attenuate Methamphetamine-Induced Microglia Reactivity and Enhance Neuronal Plasticity
Source: Cells. 2022 Jan 21;11(3):355. doi: 10.3390/cells11030355 (PMC8834016; doi:10.3390/cells11030355)
Supplement: Supplementary file 1 [file cells-11-00355-s001.zip › cells-1530608-supplementary.pdf]

Supplementary Information for

"Neuron–Microglia Contact-Dependent Mechanisms Attenuate Methamphetamine-Induced Microglia Reactivity and Enhance Neuronal Plasticity" by Bravo et al.

Contents within the present file

1. **Supplementary Tables:**

   Supplementary Table 1 - Antibodies for immunocytochemistry

   Supplementary Table 2 - Primer sequences used in qRT-PCR

   Supplementary Table 3 - Antibodies for flow cytometry

2. **Supplementary Figures and Legends:**

   Supplementary Figure S1 and respective legend

   Supplementary Figure S2 and respective legend

1. Supplementary Tables

Supplementary Table S1. Antibodies for immunocytochemistry

Antibody	Dilution	Company
**Anti-Arginase 1**	1:100	Santa Cruz Biotechnology
**Anti-β3-tubulin**	1:1000	Biolegend
**Anti-GFAP**	1:500	abcam
**Anti-Iba1**	1:500	Wako
**Anti-iNOS**	1:200	Santa Cruz Biotechnology
**Anti-PSD95**	1:500	Life Technologies
**Anti-VGlut1**	1:1000	Synaptic Systems
**Anti-GAD67**	1:100	Santa Cruz Biotechnology
**Anti-Tyrosine hydroxylase**	1:200	Merck Millipore
**Anti-mouse Alexa 488**	1:1000	Life Technologies
**Anti-rabbit Alexa 568**	1:1000	Life Technologies
**Anti-goat DyLight 650**	1:1000	abcam

Supplementary Table S2. Primer sequences used in qRT-PCR

Gene	Forward Sequence	Reverse Sequence
**TNF**	TTCTCATTCCTGCTTGTGGC	CTACAGGCTTGTCACTCGAATTT
**Il1-β**	AGGAGAACCAAGCAACGACA	CTTGGGATCCACACTCTCCAG
**Il-6**	GGAGCCCACCAAGAACGATA	CAACTGGATGGAAGTCTCTTGC
**Il-10**	GTTGCCAAGCCTTATCGGAA	GAAATCGATGACAGCGCCT
**TGF-β**	TGGAAATCAACGGGATCAGC	AGTTGGTATCCAGGGCTCTC
**CD200**	ATGGGCAGTCCGGTATTCA	AAGGATGCAGTTGTGTGCAG
**CD200R**	CTTCGCGGCTGAGTCAAGTT	AGGCTGTCCTCTGAGGGTTA
**CD22**	CTAGGCACGGGAAAGTCACC	CTGGCCACTGGAATTGTCCT
**CD45**	AACAACCGACGATGGTGCC	ACGCACAGTAACGTTCCCAA
**CX3CL1**	CCATCATCCTGGAGACGAGA	GCCGAGGTGATCCTAGGTGT
**CX3CR1**	GCCCTTCTGGACTCACTACC	TGCACTGTCCGGTTGTTCAT
**CD95**	TATCACTGCACCTCGTGTGG	GTTGCCTCTTCCGGTACCTT
**S18**	CTTCCACAGGAGGCCTACAC	ACCACATGAGCGTATCTCC

Supplementary Table S3. Antibodies for flow cytometry

Antibody	Dilution	Company
**APC anti-rat CD11b/c**	1:100	Biolegend
**Pacific Blue™ anti-rat CD45**	1:100	Biolegend
**FITC anti-rat CD200 Receptor**	1:50	Biolegend
**Anti-CD95**	1:100	abcam
**Anti-mouse Alexa 488**	1:1000	Life Technologies

2. Supplementary Figures and Supplementary Legends

Supplementary Figure S1

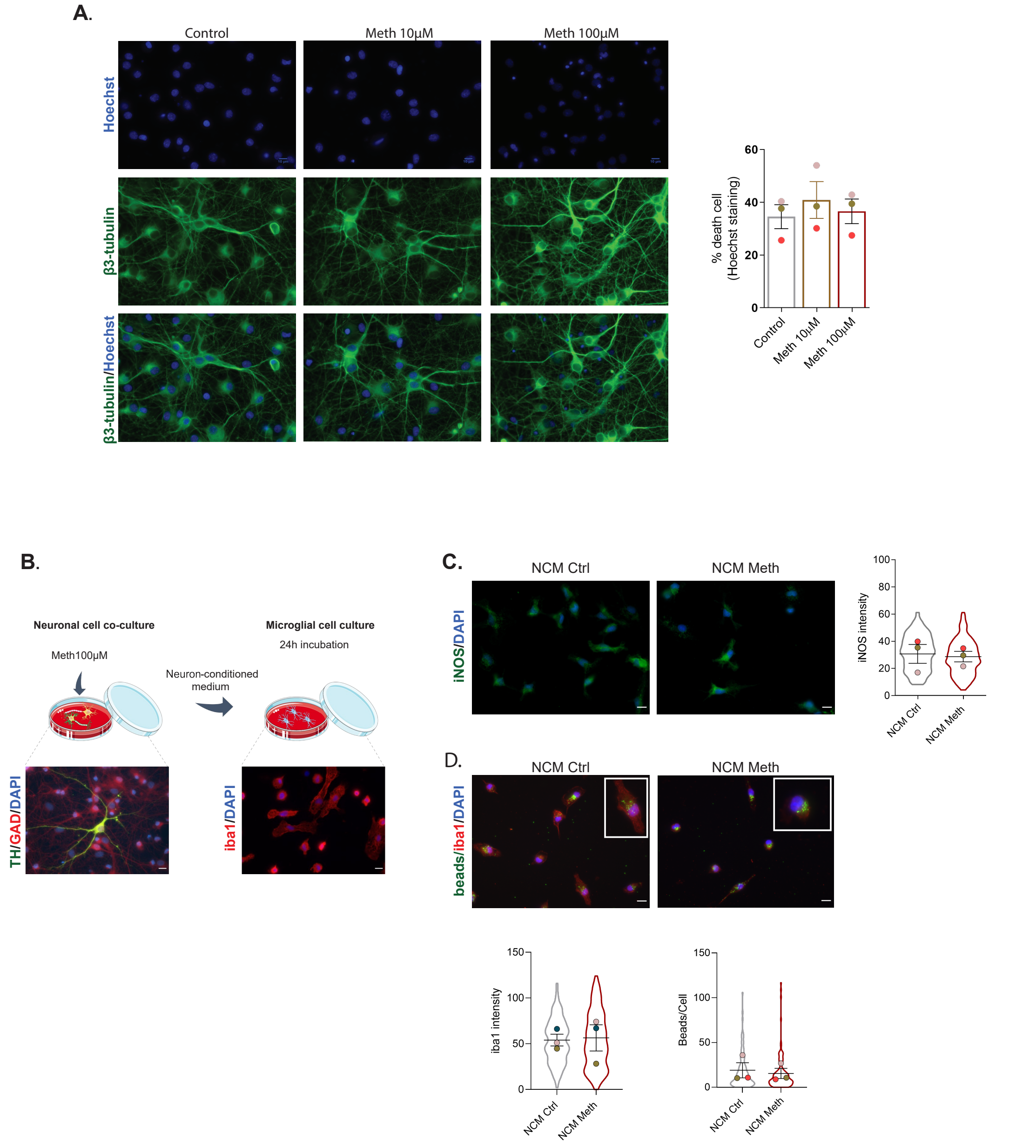

Supplementary Figure S1. **(A)** Fluorescence imaging of microglia cells labeled for β3-tubulin (green) and Hoechst (blue) incubated with Meth 10 and 100 µM for 24 h. Results are expressed as mean ± SEM of the percentage of neuronal death for three independent experiments. Statistical analysis was performed using one-way ANOVA, followed by Tukey multiple comparisons test. **(B)** Neuronal cell cultures from striatum and mesencephalon were incubated with 100 µM Meth for 24 h. Following that, primary microglia cell cultures were incubated for 24 h with the conditioned media obtained from neurons treated with Meth (NCM Meth) or from control neuronal cell cultures (NCM Ctrl). **(C)** Fluorescence imaging of microglia cells immunolabeled for iNOS (green) incubated with NCM Ctrl or NCM Meth for 24 h. Results express the iNOS

intensity (average ±SEM) of three independent cultures (NCM Ctrl – 146 cells; NCM Meth – 178 cells). **(D)** Fluorescence imaging of microglia cells immunolabeled for iba1 (red) incubated with microbeads (green) and treated with NCM Ctrl or NCM Meth for 24 h. Results express iba1 intensity and the number of beads per cell (average ±S EM) of three independent cultures (NCM Ctrl – 142 cells; NCM Meth – 131 cells). Symbol colors represent the mean of each independent cell culture and the violin plots the variability of all cells quantified. Statistical analysis was performed using a linear mixed model followed by Tukey–Kramer comparison test. Scale bar 10 µm.

Supplementary Figure S2

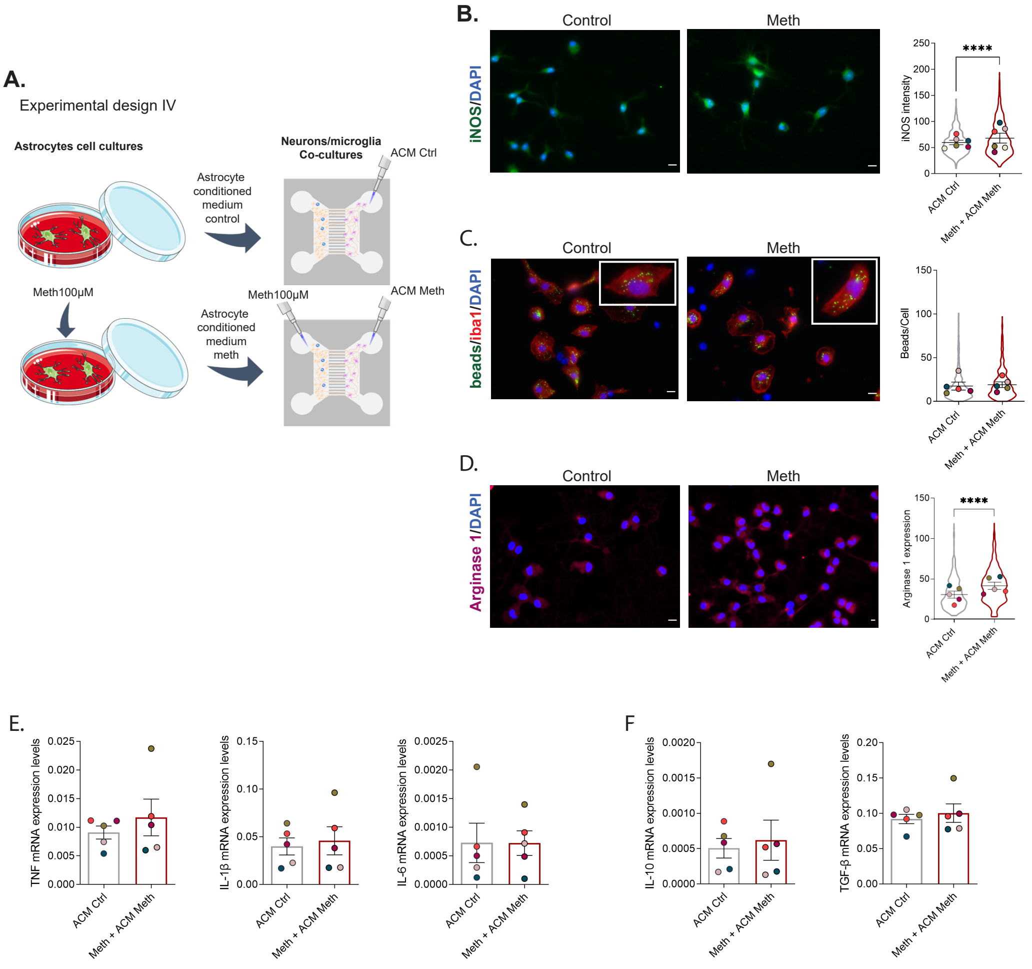

Supplementary Figure S2. (A) Experimental design IV – Primary cell cultures of astrocytes were incubated with Meth 100 µM for 24 h. Neuronal cells from the hippocampus were seeded in one side of a microfluidic device, the axons grew to the other compartment for 12 days and microglia cells were seeded in the axonal compartment. The conditioned media obtained from astrocytes treated with Meth (ACM Meth) or from control astrocytes cultures (ACM Ctrl) was added to the

microglia/axonal compartment and Meth 100 μM was added to the neuronal compartment for 24 h. **(B)** Fluorescence imaging of microglial cells co-cultured with neurons and exposed to ACM Ctrl or ACM Meth + Meth for 24 h. Microglia cells were immunolabeled for iNOS (green) and results express the iNOS intensity (mean ±SEM) of five independent cultures (ACM Ctrl – 480 cells; ACM Meth + Meth - 459 cells). **(C)** Fluorescence imaging of microglia cells co-cultured with neurons and exposed to ACM Ctrl or ACM Meth + Meth for 24 h. Microglia were incubated with microbeads (green) and immunolabeled for iba1 (red). The results represent the number of beads per cell (mean ±SEM) of five independent cultures (ACM Ctrl – 460 cells; ACM Meth + Meth - 430 cells). **(D)** Fluorescence imaging of microglial cells co-cultured with neurons and exposed to ACM Ctrl or ACM Meth + Meth for 24 h. Microglia cells were immunolabeled for arginase 1 (magenta) and results express the arginase 1 intensity (mean ±SEM) of six independent cultures (ACM Ctrl – 395 cells; ACM Meth + Meth - 403 cells). Symbol colors represent the mean of each independent cell culture and the violin plots the variability of all cells quantified. Statistical analysis for B, C and D was performed using a linear mixed model followed by Tukey–Kramer comparison test (****$p<0.0001$). Scale bar 10 μm. **(E)** RT-qPCR for TNF, Il-1β and Il-6 from microglia and neurons co-cultured cells in a microfluidic device, where microglia were exposed to ACM Ctrl or ACM Meth + Meth for 24 h. **(F)** RT-qPCR for Il-10 and TGFβ from microglia and neurons co-cultured cells in a microfluidic device, where microglia were exposed to ACM Ctrl or ACM Meth + Meth for 24 h. In both cases, results were normalized to the S18 gene and are expressed as the mean ±SEM of five independent cultures. Symbol colors represent the mean of each independent cell culture. Statistical analysis was performed using paired Student's *t*-test.
